# “Small Journals”, indexes and ecancer: an opportunity for Latin America

**DOI:** 10.3332/ecancer.2013.ed30

**Published:** 2013-12-02

**Authors:** Herbert Stegemann

**Affiliations:** Psychiatry Department, Hospital Vargas de Caracas, Venezuela

In terms of research and the publications resulting from it, there is no uniformity in the amount of production in the Latin American region. Argentina, Brazil and Mexico are great generators followed with a large gap by Chile, Colombia, Cuba, Venezuela and other countries. Most biomedical printed journals originating in the region have a particular characteristic which is that they can be grouped under the title “Small Journals”. The translation of the phrase into Spanish seems very simple, however, due to its unique characteristics clear criteria have not yet been defined. 

By the end of 2006, the World Association of Medical Editors (WAME) [1] decided to create a “Small Journals Task Force” with specific goals. It was inspired by the fact that worldwide there is a substantial difference between the classic prestigious international well-established biomedical journals and a large group of “weak” journals with very different needs and profiles. There then followed an initial dilemma which consisted of defining a “Small Journal”. Many elements came into play: financial, circulation, distribution, publication frequency and regularity, punctuality, language, quality of content, human resources, arbitration process, structure and content, visibility and presence in libraries, indexes and databases, Impact Factor, etc. Unfortunately, the specific Latin American scientific publishing reality is underrepresented in WAME. It is not known precisely how many biomedical journal titles exist in the region, but there are certainly several hundred. Some of these publications have a valuable historical tradition to the point that many have over fifty years of circulation and others have been publishing for over a century on a more or less regular basis. 

If we turn to an Internet search or to well-known organizations (Council of Science Editors [2], European Association of Science Editors [3], the International Committee of Medical Journal Editors [4], World Association of Medical Editors [1]), we will find that no clear definition for this group of publications has yet been achieved, although the term is widely used. Most of the journals in this region are weak from the viewpoint of management and finance but their editors make great efforts to get as close as possible to meeting recognized international standards of quality and scientific content, including the peer review process. Lack of regularity is perhaps the main common denominator. Within this scenario researchers and authors in Latin America are faced with the decision of where to place their manuscripts to see them printed and referenced. On the one hand, you want to strengthen and enhance regional journals with quality articles but with little or no chance of international exposure, and on the other hand, there is the temptation of being able to see the results of your work in a “classic” journal with high visibility and Impact Factor. The decision for the authors is not easy. There are many factors involved: the need for academic and peer recognition, career advancement opportunities, admission to prestigious institutions, funding for future research or financial backing for articles in open access journals, among others. 

During recent years, there has been some progress in the process of the internationalization of Latin American periodicals with articles or at least titles and abstracts in English as well as electronic versions. We even have virtual or electronic databases and libraries, among which we should mention the prestigious BIREME [5] and SciELO [6], which have allowed us to develop our own scientometrics tools. 

In a documented review [7], Delgado-Troncoso makes a thorough analysis of open access journals in Latin America and the Caribbean, and concludes that this group of journals and their inclusion in SciELO (Brazil, 1997) and RedALyC (Mexico, 2002) [8] has provided significant increased visibility for the respective authors both within their own countries and in the entire region, as well as globally, with clear exponential growth rates. 

These, and other indices and regional databases arose from the need to achieve a kind of window on the world view of the limited representation achieved with other resources such as the highly successful PLoS (USA, 2000) [9]. It is emphasized that the absence of a clear government policy influences the lack of or reduction in scientific production in some countries. 

To summarize, there was a clear need to improve adherence to mainstream international quantitative criteria and to overcome the high costs of subscription journals characterizing high international scientific prestige. It is a breakthrough that has required poorly recognized but huge individual effort, often without any financial compensation or academic recognition. 

A clear horizon still looks distant. Many of the countries in Latin America lack well-defined policies in this area. Moreover, there is still significant segregation between the various countries in the region. Although meetings and scientific conferences are held, the possibility for exchanging information on references, authors and peer reviewers is remote [10]. 

Recently, another option for Latin American authors specifically in the field of oncology has been developed: *e*cancer [11]. This is a free electronic resource, fully peer reviewed, with recognized scientific quality and following up to date publication methods. The website contains various media elements which open the doors to the scientific and academic world and ensure immediate and wide visibility and exchange of information, not only at the national but at the regional and international level as well. It is worth mentioning that the journal accepts articles in Spanish, Portuguese and English with free translation provided to accepted articles. *e*cancer is a valuable means of bringing global awareness to the various specific scientific, social, cultural and economic features that characterize Latin America. 

## Notes

Watch Dr Stegemann discuss this important issue (in Spanish).
